# Unveiling the Multifaceted Problems Associated with Dysrhythmia

**DOI:** 10.3390/ijms25010263

**Published:** 2023-12-23

**Authors:** Adrianna Witczyńska, Aidas Alaburda, Grzegorz Grześk, Jacek Nowaczyk, Alicja Nowaczyk

**Affiliations:** 1 Department of Organic Chemistry, Faculty of Pharmacy, Collegium Medicum in Bydgoszcz, Nicolaus Copernicus University, 87-100 Toruń, Poland; adrianna.witczynska@doktorant.umk.pl; 2 Department of Neurobiology and Biophysics, Institute of Bioscience, Vilnius University Saulėtekio Ave. 7, LT-10257 Vilnius, Lithuania; aidas.alaburda@gf.vu.lt; 3 Department of Cardiology and Clinical Pharmacology, Faculty of Health Sciences, Collegium Medicum in Bydgoszcz, Nicolaus Copernicus University, 87-100 Toruń, Poland; g.grzesk@cm.umk.pl; 4 Department of Physical Chemistry and Physicochemistry of Polymers, Faculty of Chemistry, Nicolaus Copernicus University, 7 Gagarina St., 87-100 Toruń, Poland; jacek.nowaczyk@umk.pl

**Keywords:** dysrhythmia, electrobiological techniques, pharmacotherapy, patch clamp

## Abstract

Dysrhythmia is a term referring to the occurrence of spontaneous and repetitive changes in potentials with parameters deviating from those considered normal. The term refers to heart anomalies but has a broader meaning. Dysrhythmias may concern the heart, neurological system, digestive system, and sensory organs. Ion currents conducted through ion channels are a universal phenomenon. The occurrence of channel abnormalities will therefore result in disorders with clinical manifestations depending on the affected tissue, but phenomena from other tissues and organs may also manifest themselves. A similar problem concerns the implementation of pharmacotherapy, the mechanism of which is related to the impact on various ion currents. Treatment in this case may cause unfavorable effects on other tissues and organs. Drugs acting through the modulation of ion currents are characterized by relatively low tissue specificity. To assess a therapy’s efficacy and safety, the risk of occurrences in other tissues with similar mechanisms of action must be considered. In the present review, the focus is shifted prominently onto a comparison of abnormal electrical activity within different tissues and organs. This review includes an overview of the types of dysrhythmias and the basic techniques of clinical examination of electrophysiological disorders. It also presents a concise overview of the available pharmacotherapy in particular diseases. In addition, the authors review the relevant ion channels and their research technique based on patch clumping.

## 1. Introduction

The term *dysrhythmia* is used to describe an abnormal heartbeat [1], but it can also be used in relation to conduction irregularities in other organs, such as those of the digestive system (gastric dysrhythmia), the brain (brain dysrhythmia), muscle, or eye [2].

Cardiac dysrhythmia (currently called arrhythmia) is a common disorder that manifests as an abnormal or irregular heartbeat. An abnormal heart rate means that the heart is beating too fast (usually over 100 beats per minute) or too slowly (usually less than 60 beats per minute) [3].

Gastric dysrhythmias are defined as abnormal myoelectrical rhythms of the stomach. They are classified as tachygastria, bradygastria, and normogastria [4,5]. Gastric dysrhythmias may cause nausea, dyspepsia, etc.

**Figure 1:**
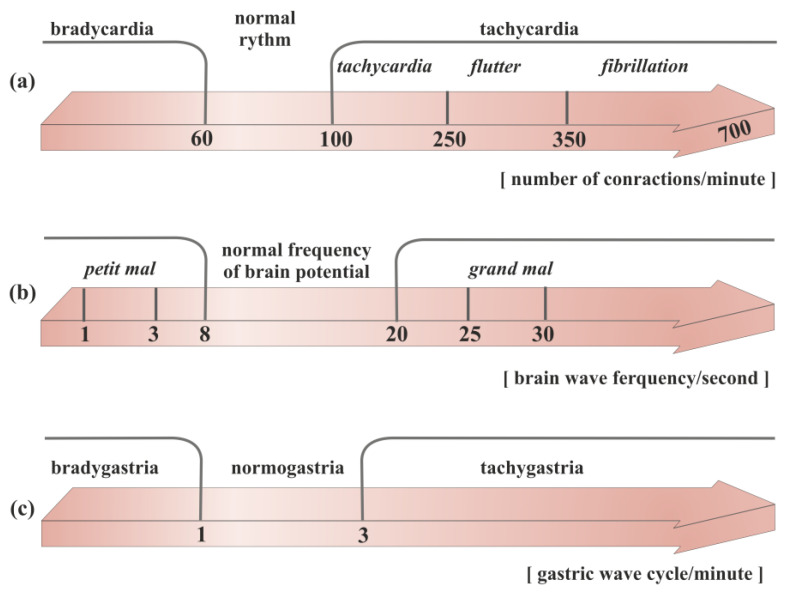
Division of the rhythm of propagation of electrical waves in different organs of the body: (**a**) heart, (**b**) brain, (**c**) stomach.

The normal frequency of brain potentials is around 8–20 per second (Figure 1) [6]. During seizures, the normal electrical pattern is disrupted by sudden and synchronized bursts of electrical potentials that may briefly affect consciousness, movements, or sensations [7,8,9]. Therefore, seizures can be considered as a form of brain dysrhythmia.

Among the most important pathomechanisms of various dysrhythmias are disturbances in the conduction of electrical impulses in various organs and tissues. This was chosen as the canvas for our review.

In order to study and monitor electrophysiological waves, modern medicine uses a variety of imaging techniques. The most common among these are electrobiological measurements that allow imaging of the electrical activity of different organs. These techniques include electrocardiography (ECG) designed to monitor heart rates, electromyography (EMG) to measure muscle contractions, electroencephalography (EEG) and magnetoencephalography (MEG) to study brain waves, electrogastrography (EGG) used to imaging stomach electrical activity, and electrooculography (EOG) dedicated to measuring eye dipole fields [10,11,12].

Recent evidence suggests that dysrhythmias may be interrelated and influence one another [13,14]. The heart, stomach, intestines, and brain have normal and abnormal rhythms. The aim of this paper is to familiarize the reader with the latest research on dysrhythmia and its consequences for treatment. In general, dysrhythmia results from different origins; the three most prominent include electrical, structural, and genetic factors. In the literature on the topic, most focus is directed to the genetic factor. One can find a plethora of excellent works evaluating channelopathies at different levels of abstraction, starting from the molecular level up to macroscopic therapeutic aspects. The subject of this work, however, does not concentrate on the genetic origins of dysrhythmia. The literature lacks an approach to this issue, emphasizing the similarities and relationships between electrical processes in different tissues. For this reason, the authors have attempted to discuss the phenomena occurring in the nervous, digestive, and heart systems. This review includes an overview of the types of dysrhythmias and the basic techniques of clinical examination of electrophysiological disorders. It also presents a concise overview of available pharmacotherapies in particular diseases. In addition, the authors review the relevant ion channels and their research technique based on patch clamping.

## 2. Short Overview of the Types of Dysrhythmias

### 2.1. Cardio Arrhythmia

The term arrhythmia has been in use for many years. It appears in this form even in the titles of textbooks and manuscripts. However, the strict meaning of it is no rhythm. Primarily, the word was meant to indicate the lack of a “correct” rhythm (Figure 1). Due to doubts, recently the term dysrhythmia is increasingly being used. It should be emphasized, however, that arrhythmia is still a word assigned to cardiology.

**Figure 2:**
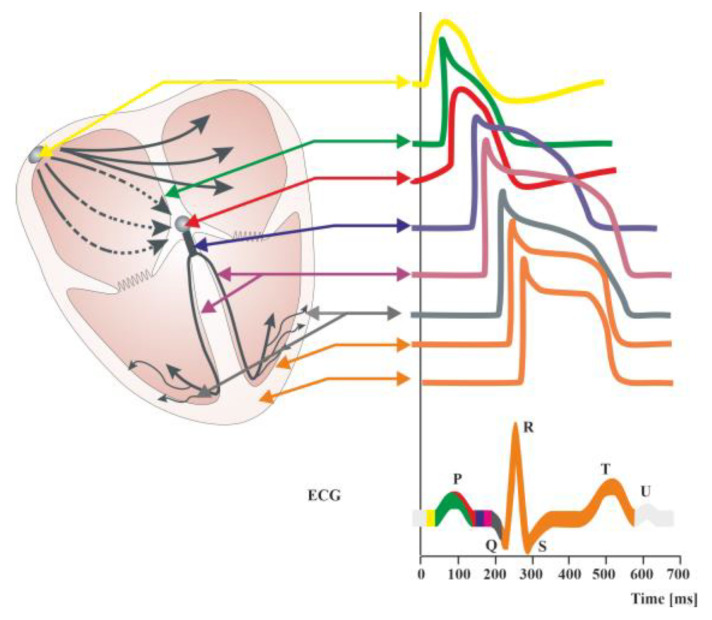
Diagram of the distributions of action potentials in individual cells of the cardiac stimulus–conduction systems. Summary changes in cardiac action potential are recorded in the form of an electrocardiographic curve (ECG). ECG showing the course of electrical phenomena during normal cardiac activity (healthy heart). The ECG obtained from the surface of the skin is the sum of the potentials conducted in the working heart. It is possible to record potentials in individual structures of the conduction system of the heart during electrophysiological examination. The QT interval includes the duration of depolarization (QRS complex) and repolarization (ST segment and T-wave) of heart muscle cells. A normal QT interval is defined as less than 450 [ms] in men and less than 460 [ms] in women [9]. Prolongation of the QT interval (LQTS syndrome) is indicative of a slowing of the repolarization process, meaning that the return to the resting membrane potential value after the depolarization of cardiomyocytes is delayed. This favors the occurrence of polymorphic ventricular tachycardia of the torsade de pointes type clinically referred to as torsade de pointes ventricular arrhythmia (TdP), which can cause syncope and sudden cardiac death in healthy, young individuals [9].

An irregular heartbeat indicates that something has interfered with the heart’s natural rhythm. However, a regular rhythm does not necessarily mean that there is no arrhythmia. There are numerous examples of abnormal heart rhythms with regular ventricular activity. They are thus more difficult to detect when relying solely on clinical symptoms. Examples include an atrial flutter with permanent conduction block, atrial fibrillation with third-degree atrioventricular block, or rhythms coming from centers lower than the sinoatrial node. This might be caused by an odd electrical signal that regulates the heartbeat. This signal could be halted by cardiac scar tissue, or it might begin too early and appear as though the heart is skipping beats. Disturbances in the electrical characteristics of the heart are known as arrhythmias. These disturbances can be split into two categories: automatic abnormalities, characterized by an automatically generated electrical impulse, and abnormalities, which occur when an impulse is conducted after it has been generated (transmission abnormality). From a different perspective, arrhythmias can be classified into tachyarrhythmias, corresponding to an accelerated rhythm, and bradyarrhythmias, referring to an abnormally slow heartbeat. (Figure 1). Moreover, heart rate variability (HRV), the fluctuation in the time intervals between adjacent heartbeats, is commonly used to characterize the interplay between sympathetic and parasympathetic systems [15,16]. It has been demonstrated that HRV parameters are frequently decreased in patients with cardiac, autonomic neuropathy, and other pathological conditions [17,18,19]. Arrhythmias are further divided into groups according to their anatomical origin, such as atrial, atrioventricular (AV), or ventricular (occurring below the bundle bifurcation) [20,21,22,23]. Paroxysmal atrial fibrillation, altered HRV, tachycardia, ventricular repolarization disorders manifested by prolonged corrected QT segment (Figure 2), changes in the morphology of the ST segment and T-wave, as well as numerous supraventricular and ventricular extrasystoles, are among the most frequently mentioned electrocardiographic disorders [24,25]. The frequency of these disorders significantly correlates with an unfavorable final outcome of treatment, not only during hospital treatment but also in the period up to a year after its completion [24].

### 2.2. Gastro Dysrhythmia

Tachygastria is characterized by an increase in the frequency of the slow wave; bradygastria occurs when the frequency is lower than normal; and arrhythmia occurs when there is no rhythmic activity, based on the dominant frequency of gastric myoelectric activity (GMA) (Figure 1). Abnormalities of the gastric slow-wave frequency (tachygastria, bradygastria, and arrhythmia) may occur spontaneously, which is associated with disturbances in normal gastric contractile activity.

Numerous human and animal investigations have demonstrated that a variety of neurohumoral variables contribute to the development of stomach dysrhythmias. Slow-wave disturbances may be brought on by antral distension and enhanced lipid supply to the intestines, which may then result in the emergence of nausea. The cholinergic and serotonergic pathways may play a role in mediating this. Similar to estrogen, progesterone can also interfere with a person’s stomach’s slow-wave pattern. Slow-wave dysfunction in diabetics and smokers appears to be mediated by an excess of prostaglandins in the gastric smooth muscle. On the other hand, the development of stomach dysrhythmias related to motion sickness is significantly influenced by the central cholinergic pathways. Vasopressin secreted by the pituitary may play a role in mediating this. Although it is challenging to pinpoint a precise causal role for slow-wave rhythm disruptions in the development of nausea and vomiting, the quest for innovative antiemetic medicines has begun based on their potential to eliminate or stop the development of stomach dysrhythmia. Prostaglandin synthesis inhibitors, central muscarinic receptor antagonists, and dopamine receptor antagonists are examples of this [26].

Gastric dysrhythmias are associated with a variety of clinical disorders, some of which may contribute to the production of nausea and vomiting. With diabetic gastroparesis, tachygastria and bradygastria can develop in up to 70% of patients [27]. Some diabetics show concurrent loss of the increase in signal amplitude typically seen with meal eating in addition to electrocardiogram rhythm abnormalities. Vomiting frequently occurs in conjunction with the occurrence of stomach dysrhythmic activity.

Several pieces of evidence point to the stomach’s ability to make its own prostaglandins as a major cause of gastric dysrhythmic activity [28].

It has long been understood that the stomach and heart are connected. Heberden initially gave a description of it in 1768. It was referred to as Roemheld’s gastro-cardiac syndrome in later years. This syndrome (AF) shows how upper GI disease can lead to chest pain that feels like angina and heart rhythm problems, such as atrial fibrillation [29,30].

It is generally acknowledged that gastroesophageal reflux disease (GERD) and non-cardiac chest discomfort are related. It is hard to tell the difference between chest pain caused by esophageal and cardiac ischemia because the heart and distal esophagus share an afferent vagal supply. Also, GERD can cause chest pain that does not come from the heart but feels like it does. This results in the misinterpretation of GERD chest discomfort as angina pectoris, and vice versa, in clinical settings. Additionally, the presence of both GERD and myocardial ischemia may make someone more susceptible to developing myocardial ischemia by tipping the sympathovagal balance in favor of the parasympathetic component. This mechanism might set off a cardiac–esophagogastric reflex, which would reduce myocardial perfusion [31].

There are at least four different ways to look at the “pro-arrhythmic” interaction between the circulatory system and the digestive tract: anatomical, neurogenic, endocrine, inflammatory, and neurogenic factors. Each of these components has the ability to alter the activity of the three main elements that lead to the development of AF: (a) the triggering stimulus (physiological and pathological automatism, triggered activity, electromechanical coupling), (b) focal conduction disorders (distribution of atrial muscle fiber refractory periods, local slowing of conduction), and (c) the creation of local circulating excitation waves and loops [32].

Vagal activity increases when the esophagus environment becomes more acidic. Therefore, acid gastric reflux may be the cause of arrhythmia events, particularly at night, including the so-called vagotonic AF. When the vagus nerve causes an irregular heartbeat, it usually shows up as changes in the heart rate and conduction velocity, as well as the circulating excitation wave becoming shorter and more spread out [33,34].

### 2.3. Neuro

Neurocardiology is a relatively new, extremely interesting, and still developing field of medical knowledge. The effects of stress and sudden emotional states of high intensity on the cardiovascular system, and particularly on heart function, continue to attract great interest [35].

The central nervous system (CNS) is a complex structure containing numerous centers that control the functions of many organs, including the heart muscle. The interactions between brain function and the heart muscle are commonly known as the brain–heart axis. Many CNS pathologies induce myocardial dysfunction [36]. Cardiac arrhythmias resulting from traumatic brain injury (TBI) can lead to significant hemodynamic failure or cause sudden cardiac death (SCD). Various types of arrhythmias are also observed in people experiencing strong emotions or stress. These relationships clearly prove a significant relationship between brain function and heart muscle function [25,37].

It is also noteworthy that 88% of individuals with acute cerebral ischemia experience substantial cardiac damage. Cardiovascular arrhythmias can occur more or less frequently in TBI patients, and their incidence varies and is highly correlated with the nature of the damage and any associated fluid and electrolyte problems. Patients with severe intracerebral bleeding are most at risk for different types of electrocardiographic abnormalities (60–70% of patients need cardiac care), whereas isolated cerebral edema increases the risk of different arrhythmias in roughly 40% of patients. It should be underlined that patients with an altered water and electrolyte balance (e.g., due to therapy or intracerebral disease) were found to experience cardiac arrhythmias significantly more often than other patients [38].

Heart arrhythmias are mostly caused by an imbalance in the activity of the sympathetic and parasympathetic systems, a rise in the level of stress hormones in the blood, and a general response of inflammation.

Indeed, both experimentally and clinically, it has been demonstrated that neuroanatomic linkages between the brain and the heart can cause cardiac arrhythmias to occur in response to brain stimulation. Additionally, seizures can cause Takotsubo syndrome (TTS) and a range of temporary cardiac consequences, such as alterations in heart rate, heart rate variability, arrhythmias, asystole, and other ECG abnormalities [37].

The links between heart and brain disease can be divided into three groups:

- Cardiovascular ischemic strokes (e.g., atrial fibrillation, valvular disease, etc.). Epidemiological data show that AF increases the risk of stroke by about 5-fold. Predisposing factors for AF include structural and electrical remodeling of the atrial structures, which plays a key role in initiating and maintaining AF. Atherial remodeling is caused by many factors, such as stretch-induced fibrosis, hypocontractility, fatty infiltration, inflammation, vascular remodeling, ischemia, ion channel dysfunction, and calcium instability. It is worth noting that these factors are also associated with stroke. Some of the fast atrial impulses are directed to the ventricles; some penetrate only the atrioventricular node, increasing its refraction; ventricular rate and irregularity are the result of implicit conduction of atrial impulses. During increased sympathetic tone, the refraction of the atrioventricular node sharply decreases, which leads to a disproportionate increase in heart rate. Platelet activation and thrombin production go up when atrial fibrillation (AF) or fast atrial rates (FA) happen. This happens more in the left atrium than in the systemic circulation. AF additionally induces endothelial dysfunction and inflammation. Thus, while a fast atrial rate increases thromboembolic risk, AF may increase it even more [39]. In addition, over the last two decades, many studies have indicated the relationship between AF and cognitive functions. AF is now a recognized risk factor and predictor of cognitive decline and dementia [40].
- Neurocardiological diseases are a group of genetic abnormalities that affect the nervous system and cardiac muscle. Examples of these diseases include Friedreich’s disease, myotonic dystrophy, and Kearns–Sayre syndrome. These disorders cause abnormal neuromuscular signaling and mitochondrial dysfunction, which can lead to cardiac manifestations such as arrhythmias and cardiomyopathies due to disruptions in neural signaling and mitochondrial energy deficits.
- Neurogenic heart diseases refer to conditions where brain dysfunctions have a profound impact on cardiac physiology. For instance, heightened stress or anxiety responses caused by sympathetic nervous system activation can trigger cardiac arrhythmias and affect heart rate dynamics. Additionally, neurological disorders such as epilepsy can cause Takotsubo syndrome (TTS) and a range of temporary cardiac effects that include fluctuations in heart rate variability, arrhythmia, and even asystole, as shown in electrocardiographic recordings [35,41,42,43].

### 2.4. Epilepsy

One of the best-known rhythm abnormalities that can lead to a number of disorders is brain electric dysrhythmia (Figure 1). The normal electrical activity of the brain is disrupted when seizures occur. More precisely, a clinically observed seizure is merely an external manifestation of a disturbed rhythm of brain potentials. The 2017 International League Against Epilepsy (ILAE) revised the classification of seizure types [6]. The electroencephalograph (EEG) can clearly classify rhythms into three main groups, which correspond to the three main categories of epileptic seizures. They are all characterized by a large increase in voltage during the peak of the seizure. The feature distinguishing them is the difference in the waves’ frequency. In tonic–clonic (also called *grand mal*), these waves speed up to 25–30 per second [8]. In psychomotor attacks (psychic variants), the pace slows down to 3 or 4 per second. In nonmotor (also called absence or *petit mal*), there are alternating changes at a rate of 3 per second [9]. It can be said simply that in *grand mal*, cortical activity is abnormally fast; in a psychomotor attack, it is abnormally slow; and in *petit mal*, it alternates between fast and slow [7].

The common neurodegenerative condition, epilepsy, is characterized by paroxysmal cerebral dysrhythmia. Through their influence on the autonomic system, which serves as the last effector that modifies heart activity, cortical regions and subcortical structures are connected to cardiac function (Figure 2). Additionally, feedback from the cardiovascular system can affect the autonomic outflow by activating neurocardiac reflexes. Controlling the flow of calcium, sodium, and potassium through different ion channels on the surface of cardiomyocytes is important for controlling the heart’s electrical and mechanical activity through sympathetic and parasympathetic nerves. The parasympathetic system has the opposite effect, and the sympathetic system improves heart rate (HR), repolarization, contractility, and relaxation by changing these things. Given that reduced arrhythmic thresholds are caused by increased sympathetic activity, genetic susceptibility to enhanced responsiveness to autonomic innervation is demonstrated by a variety of ion channel diseases [37].

Since epileptic people have a higher prevalence of cardiac comorbidities than the general population, seizures can cause a variety of transient cardiac effects during the pre-ictal phase, including an increase in blood pressure (BP), changes in HR, arrhythmias, and other ECG abnormalities [37,44].

**Figure 3:**
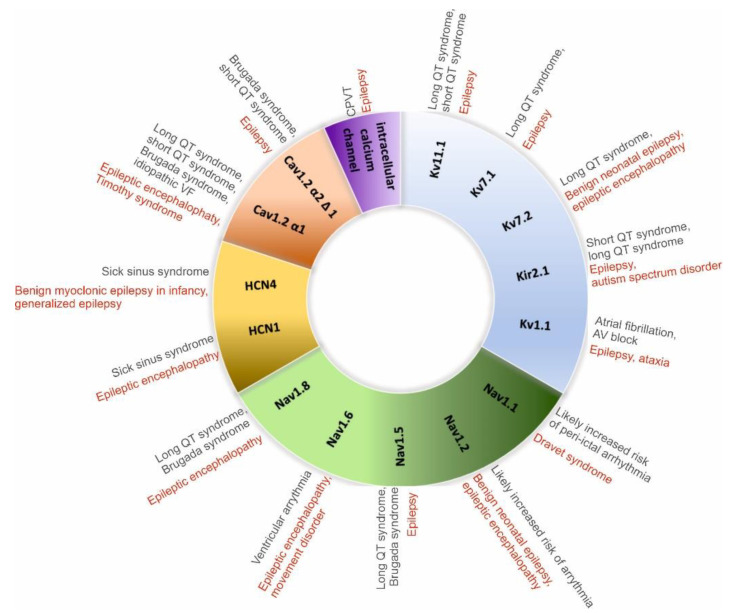
Main channelopathies associated with epilepsy and arrhythmias [35].

The risk of arrhythmias and sudden cardiac death is higher in people with a genetic predisposition to an increased response to autonomic innervation, as evidenced by a reduced arrhythmic threshold caused by increased sympathetic activity (e.g., long QT syndrome [LQTS], Brugada syndrome, catecholaminergic polymorphic ventricular tachycardia, Figure 3).

It is possible to see signs of peri-ictal arrhythmias as a result of the autonomic imbalance brought on by seizure activity. The most prevalent clinically important arrhythmia, asystole, which is typically self-limiting and has a greater prevalence in people with TLE, is more common than sinus tachycardia during the ictal phase. There are several factors that can cause asystole, sinus bradycardia, and atrioventricular (AV) block [37]. These include sympathetic activation followed by the vagal cardioinhibitory reflex, and stimulation of the limbic cortex leading to parasympathetic outflow.

### 2.5. Muscle Dysrhythmia

Neuromuscular diseases (NMDs) are inherited or acquired conditions affecting skeletal muscles, motor nerves, or neuromuscular junctions [45]. The neuromuscular junction is the site of impulse transmission between nerve terminals and muscle fibers [46]. This process mainly requires the release of presynaptic acetylcholine (ACh) and its subsequent binding to a postsynaptic ACh receptor (AChR). The result is the opening of Na^+^/K^+^ channels, which leads to muscle contraction. Synaptic vesicles containing ACh are released from the presynaptic membrane after an action potential activates voltage-gated calcium channels (VGSCas), allowing calcium influx into the nerve terminal [47].

In mammals, each skeletal muscle fiber is innervated at a single site by a single myelinated motor axon [48]. Between the nerve and muscle cells is a synaptic gap about 50–100 nm wide. The EMG recording shows potential fluctuations of varying amplitude, frequency, and periodicity. Myotonic syndromes (conditions characterized by the inability to diastole immediately after skeletal muscle contraction) are the cause of low-amplitude and high-frequency electrical activity during muscle relaxation after contraction, which gradually disappears. When the muscle begins to contract, the magnitude of these oscillations’ amplitude ranges from 100 to 150 μV, while, in the state of maximum contraction, it is between 100 and 3000 μV. Primary muscle diseases (such as myositis or muscular dystrophy) cause a decrease in the amplitude of oscillations. Depending on the stage of the disease, the indicators will show different results: up to 500 μV in the initial phase, up to 20 μV in the final phase. On the EMG chart, the amplitude of potentials’ signals and their duration decrease. These indicators depend on the patient’s age and physical development. An abnormal graph can be caused by a layer of subcutaneous fat in the test area as well as blood-clotting disorders. A decrease in the amplitude of oscillations during repeated rhythmic stimulation of the muscle is a symptom of myasthenia gravis, a condition that disrupts the transmission of impulses from the motor nerve endings to the muscles [49]. Myotonic syndromes (conditions characterized by the inability to diastole immediately after skeletal muscle contraction) are the cause of low-amplitude and high-frequency electrical activity during muscle relaxation after contraction, which gradually disappears [50].

### 2.6. Eye—Optic Neuropathy

The human optic nerve is composed of 1.2 million axons of the retinal ganglion cells (RGCs), whose cell bodies are located in the inner layer of the retina inside the globe [51]. RGCs, as the sole connection between the eye and brain, are indispensable for visual function [52]. Extraocular optic neuritis is the most common neuropathy in adults (also known as demyelinating neuropathy), which localizes to the distal portion of the nerve, and in the early stages there are no changes to the optic disc. Intraocular optic neuritis is more common in children and localizes to the anterior segment of the nerve. Optic nerve neuropathy (ONN) is characterized by a group of eye diseases that result in damage to the nerve that conducts impulses from the retina to the visual centers in the brain [53]. There are two types of optic nerve neuropathy: ischemic and toxic. The disease leads to atrophy of the optic nerve, resulting in complete and irreversible loss of vision. The main cause of the changes occurring in the section of the optic nerve located outside the eyeball is a demyelinating process. Glial tissue replaces damaged myelin, which interferes with normal nerve conduction in the optic fibers [54]. It is worth noting that the demyelinating process in extraocular inflammation is analogous to the changes that occur in multiple sclerosis (MS). In both of these pathologies, there is breakdown of the myelin of the white matter of the brain and spinal cord (MS) and breakdown of the myelin sheath surrounding the optic nerve fibers. ONN is among the most devastating disorders in ophthalmology [55]. It has also been proven that ONN is epidemiologically associated with the occurrence of MS [56]. It has been estimated that, in about 20% of patients, it may be the first manifestation of MS. It has also been observed that 50% of MS patients will develop ONN during the disease. ONN is commonly seen in the early stages of MS, mainly in developed countries, and this form of ONN is usually referred to as “typical ONN” [57].

The basic factors causing optic neuropathy include infections and inflammatory processes (most common in MS), as well as ischemic changes such as hypertension, a hypercoagulable state, diabetes, hypercholesterolemia, and ischemic heart disease [58]. This toxic neuropathy is most often triggered by the abuse of alcohol (MeOH, EtOH, CH_2_(OH)CH_2_(OH)) and tobacco (also known as tobacco–alcohol optic neuropathy) [59]. Additionally, it has been proven that B vitamins (mainly B1, B9, B12), together with folate deficiencies and the cyanide in tobacco, may be associated with the demyelination of the optic nerves [60]. Another cause of toxic neuropathy is nutritional deficiencies, as well as the use of certain drugs with high doses [61]. The drugs responsible for ONN include antimicrobial agents (linezolid, ciprofloxacin, cimetidine, and chloramphenicol), antituberculotic drugs (isoniazid and ethambutol), halogenated hydroquinolones, benzofuran derivatives (amiodarone), antiepileptic agents (vigabatrin), cGMP-specific phosphodiesterase type 5 inhibitors (sildenafil, tadalafil, and vardenafil), methotrexate, cisplatin, carboplatin, 5-fluorouracil, vincristine, cyclosporine, tamoxifen, and disulfiram, as well as tumor necrosis factor-alpha (TNF-α) antagonists (etanercept, infliximab, and adalimumab) [62]. The prognosis for drug-induced neuropathy is better than that for alcohol–tobacco neuropathy. Early discontinuation of the drug gives a chance for improvement in vision, although improvement may only occur after a long time (even after a year).

## 3. Electrobiological Techniques as Methods for Imaging the Electrical Activity of Organs

Electrobiological techniques have become a valuable asset, providing unparalleled understanding of the inner workings of organs at cellular and subcellular levels. These techniques allow scientists and physicians to explore the electrical activities of organs, leading to advancements in diagnosis and treatment and a better understanding of the intricate relationship between electrical signals and physiological processes. When analyzing various electrobiological techniques, the frequency of individual currents deserves attention. The brain waves have amplitudes ranging from approximately 1 μV to 100 μV at the surface of the scalp. The frequencies measured by the EEG range from approximately 1 Hz to 30 Hz, the dominant frequency depending on a person’s mental state. EMG signals have the same frequency range as EEG and an amplitude range of 0.2 to 2000 μV. The EOG range of frequencies is relatively low, from 1.1 to 6.25 Hz, while the amplitude is higher than that of the EEG, ranging from 1 to 4 mV [11,12].

### 3.1. ECG

The hyperadrenergic state that characterizes seizures constitutes a significant and well-established component of arrhythmia risk. Seizures have been linked to fairly severe problems with the heart’s rhythm, conduction, and repolarization. These include bundle branch block, ST-segment changes that show myocardial ischemia in 40% of seizures, and T-wave inversion. The occurrence of bradycardia and asystole in some cases may reflect the indirect influences of seizure-related respiratory disturbances and hypoxia. Ictal sinus tachycardia, an indicator of increased sympathetic activity and/or reduction in cardiac vagal tone, was reported in 82% of patients surveyed in a meta-analysis. It is known that neural influences have a big impact on heart rhythm and that many signs of autonomic function show big changes. This makes it possible to think that the neural activation of cardiac arrhythmias during epileptic seizures may play a big role in sudden cardiac death in people with epilepsy. Irregular HRV patterns suggest an altered autonomic function in epilepsy patients, indicating a propensity for increased sympathetic activity. Abnormal HRV has been implicated as a marker of increased risk for SUDEP, as it is for sudden cardiac death. Increased cardiac sympathetic nerve activity during seizures is indicated by persistent surges in heart rates to above 150 beats/min and by reduced HRV. One-third of people with drug-resistant epilepsy have heart repolarization problems, such as QT interval prolongation and dispersion, which are common ECG signs of ventricular arrhythmias [63]. High levels of T-wave alternans (TWA), a known sign of a higher risk of lethal arrhythmias and sudden cardiac death in the cardiac population, have been consistently seen between episodes in people with chronic epilepsy. It was discovered that ventricular late potentials on signal-averaged electrocardiograms (SAECG), which show ventricular depolarization and the functional substrate for ventricular tachycardia, were linked to a longer seizure duration, being refractory, and having more seizures. Abnormal cardiac conduction distinguished patients with epilepsy who died unexpectedly.

It is worth emphasizing that, at the beginning of the century, the correct assessment of HRV was difficult due to the low effectiveness of programs. It was necessary to manually select fragments and analyze them. Due to the huge amount of work involved, such an assessment was rarely used. Currently, the assessment of HRV is common and is carried out automatically during virtually every ECG and Holter test. It is also the basis for the operation of software devices such as smartwatches during rhythm analysis.

### 3.2. EEG

The recording of brain activity using an EEG is aimed at assessing the bioelectrical activity of the brain, especially the state of the nervous system. It is a non-invasive method of recording the bioelectrical activities of the brain. Nowadays, advanced electroencephalographs are used for this type of examination. During the test, a special mesh is applied to the head of the subject in places where electrodes are inserted. The whole process involves determining the changes in electrical potentials sent out by neurons. An important task is to record the action currents produced by nerve cells. Different frequencies and amplitudes of waves and rhythms make up the EEG record. Among the waves, alpha, beta, theta, delta, and sharp waves are distinguished. EEG is considered a diagnostic method, for example, in patients with epilepsy, in a coma, or with craniocerebral injuries. EEG is often counted as one type of biofeedback. Biofeedback, or biological feedback, is the return of information about changes in a person’s physiological state. These changes are usually monitored by analog measuring devices or computer-guided digital equipment.

The differences in electrical potentials are due to summed postsynaptic graded potentials from pyramidal cells, which form electrical dipoles between the body of the neuron (soma) and the branches of the neuron (apical dendrites). The flowing electrical current in the brain consists mainly of Na^+^, K^+^, Ca^2+^, and Cl^−^ ions, which are pumped through channels in the membranes of neurons in a direction regulated by the membrane potential [10].

### 3.3. EGG

Abnormal gastric electrical activity is also thought to be contributory and has historically been measured using an EGG [51]. The EGG is based on the same fundamental principles as the ECG [64]. EGG is performed under fasting and feeding conditions. During fasting, the EGG signal is typically of low amplitude. The frequency transiently decreases, and the amplitude of the signal increases after eating, presumably caused by increases in slow-wave amplitude and the generation of action potentials [26]. This technique records gastric myoelectric activity (GMA), which comprises low-frequency slow waves and neuronal spike activity. Slow waves originate from the interstitial cells of Cajal (ICCs), which show a specific distribution, arrangement, and cell shape depending on their location within various regions and tissue layers of the gastrointestinal tract. In healthy individuals, the frequency at which the power spectrum of slow waves with the highest power occurs is approximately three times per minute; this is termed the dominant frequency (DF) [65,66].

Secondly, more sophisticated methods for reliably assessing gastric electrophysiology in vivo have recently emerged, incorporating multi-electrode (high-resolution; HR) mapping. An important and consistent finding of HR mapping studies has been that novel biomarkers for altered gastric electrophysiology appear to exist in the form of patterns of gastric dysrhythmias, but that these have been inaccessible using previous EGG methods. Building on the new understanding of gastric physiology provided by serosal recordings, novel HR mapping techniques on the skin’s surface (termed body surface mapping) could offer new non-invasive opportunities to realize clinically actionable biomarkers for altered electrophysiology in gastric functional and motility disorders [67].

### 3.4. EMG

The electrical signals of the muscle cells in the human body are referred to as myoelectric signals. EMG is the method for obtaining and recording myoelectric signals in the human body. As a result of the temporal and spatial summation of the action potentials of all the active motor units of the muscle under test, located in the area of electrode recording, an electromyographic signal caused by ion flow is produced and recorded from the skin surface. The amplitudes of the recorded signals are measured in microvolts and are assumed to be in the range of 100 µV to 5 mV. In order to measure signals with such low amplitudes, they must first be amplified [68,69].

EMG assesses the function of the muscular system as well as the peripheral nervous system. This electrophysiological study is helpful in the diagnosis of many neuromuscular diseases, as it allows one to localize pathological changes in the muscles and determine their size and nature, as well as determining the dynamics of the disease process in the examined muscle. Therefore, electromyographic examination can be used to diagnose, for example, muscular dystrophies, amyotrophic lateral sclerosis, or spinal muscular atrophy. EMG is also used to diagnose diseases in the course of which nerve damage has occurred due to nerve compression (e.g., carpal tunnel syndrome, tarsal tunnel syndrome), as well as diseases of the neuromuscular junction, such as myasthenia gravis [68,69].

### 3.5. EOG

The electrophysiological testing of the eye consists of recording a bioelectric signal that is generated in the eyeball and transmitted to the part of the brain responsible for vision. Clinical electrophysiological examination is carried out using electrodes placed on the surface of the eyes, periorbital skin, or scalp. The guidelines define the basic principles of visual electrodiagnostic procedures and were developed by the International Society for the Clinical Electrophysiology of Vision (ISCEV) [70]. Those procedures include a full-field electroretinogram (ERG), a standard electroretinogram (ERG or PERG standard), a multifocal electroretinogram (multifocal ERG or mfERG), an electrooculogram (EOG), and visual cortical evoked potential (VEP). Their most recent publications are listed on the ISCEV website [63] and are freely accessible. The ERG measures the electrical response from the photoreceptor and inner retina. It has become a mainstay in evaluating retinal function in both clinical and research ophthalmology. The standard technique, full-field flash ERG using alternating current (AC) recording, commonly involves the utilization of a three-electrode system, including a corneal contact electrode, a skin reference electrode, and a ground electrode. The potential difference between the corneal electrode and the reference electrode records the summed electrical activity across the corneal surface [71,72]. The VEP represents the response from ganglion cells to the occipital cortex. Analyzing the cortical activity in response to flash or pattern stimulus allows one to distinguish abnormal features in, or the presence of any lesion along, the anterior visual pathway. VEPs are generally used in patients with demyelinating disease to detect occult optic nerve dysfunction [73]. The EOG represents the electrical response from the outer retina (photoreceptor–RPE complex). The clinical EOG is an electrophysiological test of the outer retina and retinal pigment epithelium (RPE) in which changes in the electrical potential across the RPE are recorded during successive periods of dark and light adaptation. The EOG amplitudes are measured in microvolts (μV) [74]. The light-insensitive component accounts for the dark trough and is dependent on the integrity of the retinal pigment epithelium (RPE) as well as the cornea, lens, and ciliary body. The depolarization of the RPE’s basal membrane is what causes the slow light rise of the EOG, which is the light-sensitive component. The Arden ratio, the ratio of the light peak (LP) to dark trough (DT), is used to determine the normality of the results. An Arden ratio of 1.80 or greater is normal, 1.65 to 1.80 is subnormal, and <1.65 is significantly subnormal [74].

## 4. Pharmacotherapy

**Figure 4:**
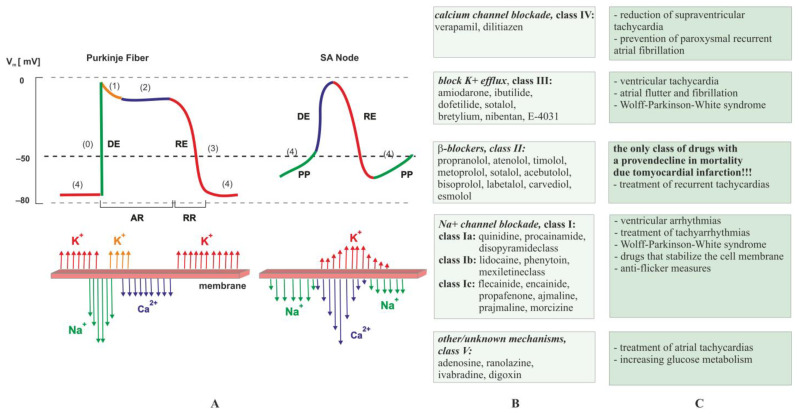
Classification scheme of antiarrhythmic drugs according to Vaughan Williams. Part (**A**) shows action potential in Purkinje fiber and SA node (where DE is depolarization, RE is repolarization, PP stands for pacemaker potential, RR is relative refractory period, AR is absolute refractory period, ↑↓ denotes flow of ions across membrane, and (0), (1), (2), (3), and (4) indicate phases of the cardiac action potential). Normally, the rate of discharge of the SA node maintains a heart rate between 60–100 bpm. Slower rates of discharge occur in the atrioventricular node (40 and 60 bpm) or Purkinje system (20–40 bpm); however, these slower rates are normally controlled by the dominant pacemaker, which has a higher intrinsic rate of discharge. Greater automaticity results in a higher rate of action potential discharge [80]. Part (**B**) presents classes and examples of antiarrhythmic drugs according to Vaughan Williams; Part (**C**) displays the clinical use of antiarrhythmic drugs.

Heart rhythm problems can be fixed by adjusting the electrical activity in the heart’s stimulus–conduction system cells and myocardial fibers in the right way [9,75,76]. Vaughan Williams in 1970 proposed a classification system of antiarrhythmic drugs (AAD), that, after slight modification, is still leading today. Drugs used in arrhythmia treatment mainly affect trans-membrane ion channels [77]. Based on the mechanism of action, the drugs can be categorized into five classes (Figure 4) [78]. Vaughan Williams’ classification, although not accounting for later advances in cardiac electrophysiology, remains the most widely used classification for categorizing AAD [79].

Classes include drugs that block sodium (class I) [79], potassium (class III), calcium (class IV) channels, β-blockers (Class II) [81,82], or compounds acting through other, unknown mechanisms (class V) (Figure 4). Each group of drugs has specific indications and side effects [83,84]. For example, sodium channel blockers are used for local anesthesia, the treatment of epilepsy, and the treatment of cardiac arrhythmias. However, complete blockage of sodium channels can result in loss of sensation, coma, or arrest of the heart, which determines the narrow therapeutic range of drugs in this group [85]. The class II and class IV antiarrhythmics mainly work on cardiac pacemaker cells, whose potential change (contains phases (4), (0), and (3)) is particularly different from that of the cardiac working cells (contains phases (0), (1), (2), and (4)). The most surprising phenomenon common to all [9,75,76,86] is proarrhythmia (the ability of a drug to induce arrhythmia) resulting from slowing of conduction or prolongation of the action potential. The proarrhythmic effect of a drug is likely when, after its use, there is a significant aggravation of existing arrhythmias or the appearance of new arrhythmias. For this reason, whenever possible, the current aim is to eliminate the causes of the rhythm disturbances. For example, ablation procedures are increasingly being performed [9,75,76]. Nevertheless, the causes of cardiovascular problems are varied; therefore, the drugs used to combat them belong to many chemical groups. If the cause of heart failure is arrhythmia, it is necessary to use antiarrhythmic drugs (Figure 4), such as amiodarone, ivabradine, propafenone, verapamil, diltiazem, or β-blockers. Cardiac arrhythmias can be caused by thyroid gland function abnormalities or electrolyte disturbances, such as potassium deficiencies, which can be supplemented by administering potassium salts or preparations containing potassium and magnesium. The cause of arrhythmias can also be the side effects of various medications, such as antibiotics, antihistamines, and psychotropic drugs. Some arrhythmias increase the likelihood of the formation of anticoagulation with direct oral anticoagulants or vitamin K antagonists [87]. In order to improve the effectiveness of treatment, reduce the risk of drug–drug interactions, or reduce the risk of bleeding complications, it may be necessary to use drug concentration-based therapy [88,89]. The guidelines of the European Society of Cardiology recommend the use of this procedure in expert centers [76].

At the turn of the century, concerns about the ventricular proarrhythmic effects of dysrhythmic drugs, along with the characteristics of electrophysiological differences between atrial and ventricular cardiomyocytes, initiated the search for AF-specific drugs. In theory, the lack of ventricular effects creates a better safety profile, allowing the simultaneous use of higher doses to increase antiarrhythmic efficacy [90].

Epilepsy, one of the oldest disorders known to man, has been treated with a variety of medications. A therapy regimen has been devised throughout history [91]. Antiepileptic drugs (AEDs) are always the first choice because many epilepsy patients experience seizure-free periods while taking an AED, despite the fact that there are various alternative treatment options for epilepsy [92], such as vagus nerve stimulation (VNS), surgery, and a ketogenic diet [93].

Several medications are available to treat epilepsy, which can lessen the frequency and intensity of seizures. According to post-traumatic epilepsy (PTE) guidelines, a single medication should be used to start treating newly diagnosed epilepsy [94]. If the therapy is ineffective or the patient does not tolerate it well, the doctor must employ an additional medication (monotherapy) or other drug combinations than those that have been previously utilized (add-on therapy). Innovative medicines provide a chance for a life devoid of epileptic seizures [91,95].

There are several generations of antiepileptic medications, including the well-known first and second generations as well as the newest third generation, which has been developing since the late 1990s. Phenobarbital, phenytoin, primidone, and ethosuximide are the oldest so-called first-generation antiepileptic medications. The following medications are considered second-generation drugs: benzodiazepines, carbamazepine, and valproinates. On the other hand, the more recent, so-called third-generation medications are vigabatrin, zonisamide, lamotrigine, felbamate, gabapentin, topiramate, phosphenytoin, tiagabine, oxcarbazepine, and levetiracetam in order of introduction to markets worldwide. The newest medications among them, with novel mechanisms of action, are styripentol, pregabalin, rufinamide, lacosamide, eslicarbazepine, retigabine, and perampanel [93,96].

In addition to being safer and more well-tolerated than the older AEDs, the newer AEDs, such as levetiracetam (LEV), oxcarbazepine (OXC), lamotrigine (LTG), topiramate (TPM), gabapentin (GP), and vigabatrin (VB), are also successful in treating a variety of seizures [93]. A very important and difficult element is the proper selection of doses. In the case of drugs of the first and second generation, it is impossible to talk about effective and safe therapy without therapeutic drug monitoring (TDM). In the case of the third generation of drugs, TDM is not necessary in most cases, although it should be considered in patients with side effects or ineffective therapy [97]. Table 1 shows AEDs classified according to their generation [98,99].

Some AEDs are classified as broad-spectrum because they treat a wide range of seizure types. Some medications are known to be ineffective for certain seizure types and, in rare cases, may worsen certain seizure types [86]. The next major determinant is the drug’s side effect profile. Preference is given to drugs with a low risk of drowsiness and cognitive dullness [100]. When choosing drugs for polytherapy, the mechanism of action is especially important because drugs with similar mechanisms of action may cause synergistic adverse effects [86,101].

AED selection is also influenced by the drug’s half-life, which is used to calculate how many doses should be taken each day, the ability to titrate quickly (lamotrigine requires a slower titration schedule, making it ineffective for use in children who frequently have seizures in intensive care units), interactions with other AEDs and oral contraceptives, and its availability in IV and oral liquid formulations. For individuals with a history of allergies, it may also be necessary to take into account the potential for drug–drug interactions [101,102].

The antiepileptic drugs carbamabyzepine, oxcarbazepine, valproic acid, phenytoyine, and lamotrigine mainly act on voltage-gated Na channels, whereas valproate and topiramate have this effect only in a part of their operation. By affecting the inactivation of Na channels, not only are the amplitude and duration of a single action potential affected, but it also reduces the ability of neurons to generate bursts of high-frequency action potentials. For this reason, the disadvantages of antiepileptic drugs are more evident at high discharge rates than at low rates (the so-called use-dependent block) [86,102].

T-type Ca^2+^ channels play an important role in the formation of absence seizures. This may explain the effects of ethosuximide and mesuximide, since these drugs lead to attenuation of T-type Ca^2+^ currents in thalamocortical neurons. Depending on the membrane potential of the neuron, due to their interference with calcium channels, they prevent the formation of low-threshold Ca^2+^ spikes and thus prevent the formation of shutdowns (absence seizures) [86]. Benzodiazepines, e.g., clobazam, clonazepam, diazepam, or lorazepam, and barbiturates, e.g., phenobarbital, are allosteric agonists of GABAA receptors and induce hyperpolarization of the cell membrane via these receptors. Felbamate blocks the glycine-binding site within the NMDA receptor. In addition, it has an inhibitory effect on the voltage-dependent Na^+^ and Ca^2+^ channels and acts similarly to the barbiturates on the GABA receptor [86,101].

Another mechanism of the antiepileptic effect of these substances is their effect on the kinetics of neurotransmitters or neuromodulators. For example, vigabatrin inhibits the enzyme GABA-transaminase and thus delays the breakdown of GABA in neurons and glial cells. By inhibiting intravascular GABA transport, vigabatrin further increases the concentration of GABA in tissue structures. In turn, tiagabine selectively blocks the uptake of GABA into neurons and glial cells [101,102].

Proton pump inhibitors (PPIs) are the most prescribed medications in the world. Originally, they were commonly the first treatment of choice for GERD. The decrease in the number of cardiac arrhythmia incidents while taking the PPIs supports the hypothesis that cardiac arrhythmias and gastroesophageal reflux illnesses are related [103]. In this case, maintenance therapy ought to be applied at a dosage that manages symptoms. The action of PPIs (pantoprazole, the most frequently used PPI) consists of inhibiting the production of hydrochloric acid by the proton pump located in the parietal cells of the gastric mucosa [104]. It is important to note that pantoprazole should be used instead of other PPIs in patients who are also on clopidogrel, because these patients are more likely to experience acute coronary events while taking other PPIs. Commercially available drugs indicated as PPIs are pantoprazole, omeprazole, esomeprazole, dexlansoprazole, rabeprazole, and lansoprazole [105]. Structurally, all currently approved PPIs are benzimidazole derivatives. Their ring structure is heterocyclic, and they have a methylsulfinyl group connecting a pyridine and a benzimidazole part. Despite the fact that all PPIs have the same basis structure and main mechanism of action, they differ in pharmacokinetic and pharmacodynamic profiles. From a chemical point of view, PPIs are lipophilic weak bases (pKa 4.0–5.0) that are membrane-permeable and acid-labile [106,107]. These medications come in a range of delivery strategies to avoid early activation and destruction by luminal stomach acid. These include gelatin capsules, coated granules given as powder for suspension, and enteric-coated tablets. Recent studies on the alleged connection between PPIs and cardiovascular illness have reignited interest in the subject [108].

Recently, it has been proven that long-term intake of PPIs might increase the risk of cardiovascular events. One of the suggested mechanisms is the effect of PPIs on the NO pathway. This effect is based on the inhibition of the enzyme dimethylarginine dimethylaminohydrolase (DDAH) by PPIs, and thus they block the degradation of asymmetric endothelial dimethylarginine (ADMA) [109]. Additionally, it has been shown that PPIs decrease the absorption of vitamin B12, leading to hyperhomocysteinemia and subsequent rises in plasma ADMA, which impair endothelial function. Additionally, it has been hypothesized that the use of PPIs reduces the amount of NO produced from dietary nitrate as a result of elevated gastric pH [63,110]. It has also been suggested that increased xanthine oxidase activity, which produces reactive oxygen species, contributes to endothelial dysfunction. The acceleration of atherosclerotic risk factors caused by PPIs has been linked to additional molecular pathways. It has been shown that esomeprazole alters gene expression, increasing the expression of the fibrinolysis inhibitor plasminogen activator inhibitor-1 and inducing telomeric shortening (an indicator of cell senescence) [108].

Drugs that impair neuromuscular transmission can block the nerve action potential or impair the release of ACh from the presynaptic membrane, such as by inhibiting the presynaptic calcium channels (1 and 2). ACh esterase inhibitors at toxic levels may increase weakness through the prolonged action of ACh. Neuromuscular transmission impairment may also result from a decreased concentration of ACh receptors or their blockage on the postsynaptic membrane [111].

Prolonged fluoroquinolone systemic therapy is associated with a higher risk of tendinitis and ruptured tendons, especially in the elderly; this side effect also occurs after therapy cessation. In addition, it can worsen muscle weakness due to an increase in a neuromuscular blockade in myasthenia gravis [112].

The basis of ONN treatment is to establish the direct cause and the possibility of its elimination. Unfortunately, this is only possible in some cases, such as compression or trauma [113]. To treat neuropathy caused by infections and inflammatory processes, steroid therapy (oral or intravenous) is helpful. However, in order to minimize loss of high-contrast vision, improve contrast sensitivity, and preserve color vision and visual field, it is important to diagnose subtypes of optic neuritis early and accurately. Rapid administration of high doses of corticosteroids and, in some cases, the use of plasmapheresis are two ways to achieve this [114,115]. The patient is given large doses of intravenous corticosteroids for the first week, after which the dose is reduced to oral corticosteroids. Therapy continues for many months, gradually reducing the dosage, after which the patient takes a maintenance dose for about 1–2 years. Intravenous administration of erythropoietin (EPO) appears to be a promising treatment for MeOH-induced ONN [116]. EPO is a cytoprotective cytokine that exerts antioxidant and anti-inflammatory effects, reduces apoptosis of nerve cells, and exhibits neuroprotective and neurodegenerative properties. Treatment of ONN induced by CH_2_OHCH_2_OH consists of the administration of fomepizole, which aims to reduce the conversion of CH_2_OHCH_2_OH to its toxic metabolites, and the use of hemodialysis [117]. Patients with alcoholic–tobacco neuropathy are given 1000 units of vitamin B12 (hydroxocobalamin) once a week for 10 weeks, and a diet rich in protein and vitamins is recommended [118]. Of course, the basic condition for the treatment is to stop smoking and drinking alcohol. Some neuropathy conditions are impossible to cure completely [119].

## 5. Patch Clamp Methods to Research Therapeutically Important Ion Channels in Excitable Membranes

Ion channels play an essential role in basic physiological functions in virtually all types of tissues and organs. They are a frequent drug target for many therapeutic agents. Ion channel dysfunctions, or channelopathy, underlie the expression of the phenotype of many serious diseases, such as cardiovascular, nervous, and respiratory pathologies [120].

Ion channel screening is now an integral part of the development and safety profiling of most new drugs currently under development. It is also an excellent tool to assess the safety profile of drugs currently in clinical use. Patch clamp methods are now considered the “gold standard” for studying ion channel function and pharmacology [26].

### 5.1. The Ion Channels Important in Dysrhythmic Disorders

There is some evidence, based on the effect of clinical drugs on cardiac APs [78], indicating the classification of cardiac ion channels into two classes. The first class contains the most important cardiac ion channels, such as K_V_11.1, Na_V_1.5, and Ca_V_1.2 [121]. The second class comprises K_V_4.3, K_V_LQT1/mink, and K_ir_2.1 and is less critical for the assessment of all drugs under the comprehensive in vitro proarrhythmia assay (CiPA) [64].

Epilepsy is a group of disorders with complex pathomechanisms, whose common feature is the occurrence of epileptic seizures. They can result in the impaired function of neurons, which comes from the defective function of various factors [85]. Potassium (K^+^) channels are present in almost every cell and in the membranes of nerve and glial cells. They are crucial for cell excitability. Currently, there are 12 channel subtypes of potassium (K_V_1 to K_V_12) [122]. The most important ion channels in the context of epilepsy treatment are Na_V_1.1–1.4 and 1.6–1.7, K_V_4 and 7 [123] Ca_V_1.2 and 2.1 [124].

Since the symptoms of GERD are closely related to gastroesophageal reflux disease, the role of acid-sensitive ion channels (ASICs) in the esophagus appears to be important [125,126,127].

Myotonia, which manifests as typical “myotonic runs” in EMG, is characterized by poor muscle relaxation after contraction [128,129]. Loss-of-function mutations in the CLCN1 gene, which codes for the skeletal muscle chloride channel ClC-1, can result in this [130]. Sodium (Na.1.4) and chloride (CLC-1) channels play an important role in muscle regulation and the therapeutic purposes of myotonia [128,131]. In mammals, there are ten subtypes of Ca_V_ channels, among which Ca_V_1.1 is specified for the excitation–contraction coupling of skeletal muscles [132,133].

TRPV1–4, TRPM8, TRPA1, or TRPC4 are some of the TRP family of channels that have been found in ocular cells. In corneal epithelial cells, epidermal growth factor (EGF) causes the release of Ca^2+^ from intracellular storage and activation of the store-operated calcium channel [134,135]. For proper vision, the retinal pigment epithelium (RPE) is crucial. RPE cells have been found to express what are known as high voltage-activated Ca^2+^ channels, according to numerous independent studies. K^+^ channels have the ability to block Ca^2+^ inflow. There must be a counterbalance for Ca^2+^ flux in RPE cells, since Ca^2+^ influx causes an increase in intracellular free Ca^2+^ and depolarization of the cell, and Ca^2+^ ions are implicated in many processes of the RPE. Because they are triggered by depolarization (voltage-gated K^+^ channels) and by Ca^2+^ itself (Ca^2+^-activated K^+^ channels), K^+^ channels make excellent candidates [136,137]. The most important subtypes of ion channels are Ca_V_1.3–1.4; Na_V_1.2; K_V_2.1–2.2, K_V_7, and K_V_8.2 [138].

### 5.2. Patch Clumping as Basic Ion-Channel Assay Methods

The abnormal behavior in rhythmicity is usually related to altered electrical activity at the cellular level. The membrane potential and its dynamics in any cell are determined by a set of specific channels. The most physiological assessment of the electrical activity of the cell is a membrane potential and measurement in current-clamp mode. The value of resting membrane potential, threshold and shape of action potential, and rhythmic activity, estimated in current-clamp mode, can pinpoint the altered electrical signaling and provide cellular correlates for abnormalities observed in ECG, EEG, EMG, and other biological measurements.

Both sharp and patch electrodes are used for recordings in current-clamp recordings. The sharp electrode with a smaller tip, if properly selected, causes less damage to the cell recorded; however, it has a limited ability to pass the electrical current. While a patch electrode with a large tip and higher current passing capacity is preferable for voltage clamp measurements, it can cause a washout of the content of a cell [139].

The membrane potential of a cell is caused by currents flowing through ion channels and charging the capacitance of the membrane. In a voltage clamp, using the patch electrode, the ion currents can be measured directly and therefore provide more precise information about the functional status of ion channels. The ion channels are characterized by activation voltage, channel conductance, dynamics of activation and inactivation, and sensitivity to pharmacological substances. While some mutations can completely disable the functionality of the ion channel, some channelopathies are related to more subtle alteration: conductance, threshold, and dynamics of activation. This functional assessment can be performed with patch clamp recordings.

A number of ion channels share the voltage range of activation; therefore, they are activated simultaneously during functional activity. This heightens the challenge of separating the ion currents recorded.

The ion channels have a different kinetics of activation/inactivation. Moreover, the majority of ion channels are voltage-dependent; their activation depends on a membrane voltage. For example, both the sodium and L-type Ca^2+^ channels activate at depolarized levels of membrane potential, sodium channels at approx. −50–40 mV, L-type Ca^2+^ at −20–10 mV. However, the kinetics of inactivation differs significantly: it is a few milliseconds for sodium channels, while L-type Ca^2+^ channels are not significantly inactivated even after a few hundreds of milliseconds. The sodium currents should be assessed immediately after the onset of a depolarizing voltage step. The holding potential of −50 mV causes the inactivation of sodium channels, and the following depolarizing steps can be used for investigating the activity of L-type Ca^2+^ channels [140].

If a few ion conductances share the same voltage range of activation, specific ion channel blockers are used. For example, sodium currents and delayed rectifier potassium currents activate at the same voltage range. Therefore, the separation of these two currents can be confirmed by TTX, a specific sodium channel blocker [140]. An inward L-type Ca^2+^ current in neonatal rat ventricular myocytes was enhanced by cesium, a non-specific blocker of the potassium channels blocker [141].

For a more detailed investigation of the properties of specific ion channels, model-expressed systems like Xenopus oocytes [142], HEK293 [143] or Chinese hamster ovarian (CHO) cells [144] are used. These cells not only express a limited selection of native ion channels, but their biochemical machinery is capable of generating functional, mature proteins from a wide spectrum of nucleic acids [145]. Therefore, the introduced ion conductances constitute the majority of electrical signals in these cells. For example, the genetic variants of KCNQ1 mutations are associated with type 1 long QT, or Romano–Ward syndrome. The functional assessment of mutated KCNQ in HEK293 cells revealed that the A300T mutation causes a left-shift in the activation voltage of these channels [142]. The patch clamp recordings provide a direct functional estimation of ion channel activity. Therefore, it is very important in identifying and explaining channelopathies, testing drugs and their side effects.

## 6. Materials and Methods

Data Selection: The analysis was performed according to the PRISMA (Preferred Reporting Items for Systematic Reviews and Meta-Analyses) statement guidelines [146].

Database Search Criteria: a combination of the key words dysrhythmia (such as cardio, gastro, neuro, muscle dysrhythmias, and optic neuropathy); electrobiological techniques (ECG, EEG, EGG, EMG, EOG); pharmacotherapy (AAD and AED); and patch clamp methods.

Databases were searched, and Science Direct, Scopus, and PubMed records were identified for full-text scientific articles published in 2019–2023 and only in chemistry, pharmacology, toxicology, or pharmacy journals. We also manually searched the bibliography of selected articles, reviews, meta-analyses, and practical tips. The authors have mutually agreed upon 146 articles. Particular emphasis was placed on the selection of types of dysrhythmias, electrobiological techniques, pharmacotherapy, and patch clamp methods.

## 7. Conclusions

The structures responsible for the movement of ions and the regulation of their balance are widely distributed in individual tissues; hence, the effects of their modulation depend on the density of their occurrence. For this reason, there are dependencies between the use of dysrhythmic treatment or the disease itself and symptoms from other organs and systems.

The cause of abnormal activity may be structural abnormalities resulting from genetic abnormalities, i.e., channelopathy. The issue of channelopathy is widely discussed in the contemporary scientific literature. However, dysrhythmia may also result from a whole range of functional abnormalities. Such disorders can also lead to significant abnormalities, e.g., seizures, in the absence of an organic change. The reason may also be drug interactions, drugs affecting the functions of channels, or stimulants. Moreover, side effects of drugs used to treat a disease affecting one system often include side effects resulting from actions in other systems. For this reason, proper pharmacotherapy is a huge challenge not only for chemists at the stage of designing in silico drugs or for pharmacologists conducting in vitro and in vivo tests, but also for clinical medicine specialists in their daily work.

Appropriate therapy should always take into account potential disease–disease, drug–disease, or drug–drug interactions. The patch clamp recordings provide a direct functional estimation of ion channel activity. Therefore, they are very important in identifying and explaining channelopathies, testing drugs and their side effects.

## Figures and Tables

**Table 1:** Generation of antiepileptic drugs.

First Generation	Second Generation	Third Generation
carbamazepine (CBZ)	felbamate (FBM)	eslicarbazepine acetate (ESL)
clobazam (CLB)	gabapentin (GPT)	lacosamide (LCS)
clonazepam (CZP)	lamotrigine (LTG)	perampanel (PER)
ethosuximide (ETS)	levetiracetam (LEV)	retigabine (RTG)
phenobarbital (PB)	oxcarbazepine (OXC)	rufinamide (RUF)
phenytoin (PHT)	pregabalin (PGB)	stiripentol (STP)
sulthiame (STM)	tiagabine (TGB)	fenfluramine (FFA)
valproic acid (VPA)	topiramate (TPM)	cenobamate (CMB)
	vigabatrin (GVG)	ganaxolone (GNX)
	zonisamide (ZNS)	
